# Microbes as medicine

**DOI:** 10.1111/nyas.15237

**Published:** 2024-10-11

**Authors:** Brendan A. Daisley, Emma Allen‐Vercoe

**Affiliations:** ^1^ Department of Molecular and Cellular Biology University of Guelph Guelph Ontario Canada

**Keywords:** live microbial therapy, microbial ecology, postbiotics, prebiotics, probiotics, synbiotics

## Abstract

Over the last two decades, advancements in sequencing technologies have significantly deepened our understanding of the human microbiome's complexity, leading to increased concerns about the detrimental effects of antibiotics on these intricate microbial ecosystems. Concurrently, the rise in antimicrobial resistance has intensified the focus on how beneficial microbes can be harnessed to treat diseases and improve health and offer potentially promising alternatives to traditional antibiotic treatments. Here, we provide a comprehensive overview of both established and emerging microbe‐centric therapies, from probiotics to advanced microbial ecosystem therapeutics, examine the sophisticated ways in which microbes are used medicinally, and consider their impacts on microbiome homeostasis and health outcomes through a microbial ecology lens. In addition, we explore the concept of rewilding the human microbiome by reintroducing “missing microbes” from nonindustrialized societies and personalizing microbiome modulation to fit individual microbial profiles—highlighting several promising directions for future research. Ultimately, the advancements in sequencing technologies combined with innovative microbial therapies and personalized approaches herald a new era in medicine poised to address antibiotic resistance and improve health outcomes through targeted microbiome management.

## Graphical abstract

The explosion of research into the human microbiome has revealed the many ways in which our microbes enhance our health. Could these host‐associated microbes be exploited as novel therapies to treat disease? In this review, the various strategies for using microbes as medicine that have been thus far employed, as well as the potential new treatments of the future, are explored and current research gaps are highlighted.

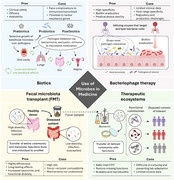

## INTRODUCTION

It has been approximately 20 years since advances in sequencing technologies started to reveal in increasing detail the microbial complexities of the human body. With this emergent knowledge, there has been a growing sense of alarm about the effects of antibiotics on the complex microbial ecosystems of the microbiome.^1^ Combined with the current antibiotic resistance crisis, this situation has driven an increasing interest in understanding how microbes can be harnessed to treat disease or improve health in the place of conventional antibiotic treatment. In this review, we have attempted to provide a comprehensive overview of established and emergent microbe‐centric therapies, tracking the increasingly sophisticated ways in which microbes are being used as medicine and indicating where the field is likely to go in the future. We start by considering *bugs as drugs* from probiotics to sophisticated microbial ecosystem therapeutics through the lens of microbial ecology as well as challenges in pharmaceutical development. We end with a summary of where we suggest that the field is headed, as well as a list of open questions and research opportunities for consideration.

## AN ECOLOGY PRIMER ON MICROBIAL INTERACTION WITHIN AN ECOSYSTEM

In order to understand how a microbial ecosystem can be manipulated effectively (i.e., through therapeutic intervention), it is first necessary to understand the microbial interactions that define the framework within which the ecosystem exists. The field is still far from understanding the intricacies of human gut microbiome ecology, although some insight has emerged from both in vivo and in vitro studies that point to the rules of engagement between microbes of this ecosystem. To date, most studies have focused on bacterial interactions within the gut microbiome, although the roles of other components, such as viruses (e.g., bacteriophages) and eukaryotes such as fungi, are increasingly starting to be included in ecological assessments.

Broadly speaking, ecological interactions can be divided into positive and negative interactions.^2^, ^3^, ^4^, ^5^ The relative importance of these in vivo interactions is subject to debate but is critical in understanding what therapeutic strategies can be developed that will influence the microbiome. Here, we will consider each category of interaction by providing examples of microbial interaction strategies and their consequences on overall gut microbial ecology.

### Positive interactions

Microbial members of the gut ecosystem are often referred to as our commensals,^4^, ^5^ literally meaning that they “share the same table” as us, their host. Ecologists define the commensal relationship as one where a given species in a partnership derives a benefit from a partner species that is, in itself, unaffected.^4^, ^5^ Although there are, undoubtedly, many true commensals within the gut community that benefit from inhabiting a host‐provided niche without contributing to the host directly, there are also an increasing number of discovered examples of host–microbial relationships within the gut that are better described as another positive type of interaction, mutualism–an interaction where both species benefit.^4^, ^5^ However, the gut microbiome consortium is not a collection of independently acting microbes, and understanding the ecosystem requires an appreciation of the many microbial interactions that occur.

Positive interactions within all microbiomes are the result of supportive activities such as cross‐feeding where one species utilizes a substrate and produces a waste product that is utilized by another.^4^ This situation is described as syntrophy.^4^ Animal‐associated microbiomes are generally highly stable ecosystems; the human gut microbiome has been demonstrated to display taxonomic stability at timescales of months or years, although most of the studies used to assess this were based on assessments of bacterial components in fecal samples.^6^, ^7^, ^8^ On the face of it, it may be thought that such stability is driven by a dominance of syntrophy between microbial members, but this is not the case. In ecology, truly syntrophic relationships tend to result in ecosystems that oscillate between high and low abundances of both partners together according to substrate availability, creating large discrepancies in abundance and destabilizing the ecosystem overall.^3^, ^5^, ^9^ Less direct forms of syntrophy where a given microbe produces a substrate that is released into the ecosystem as a public commodity for the potential benefit of all microbes present there (which, in return, work to support the commodity producer) would also seem to favorably support ecosystem stability.^3^ However, these situations tend to be overwhelmed by cheaters that utilize the public commodity substrates without giving back, eventually destabilizing the cooperation network.^3^

This is not to say that syntrophy in the human gut microbiome does not exist. Indeed, several cases have been demonstrated. For example, the bacterial species, *Bacteroides thetaiotaomicron*, has been shown to form a physical interaction with the archaeon, *Methanobrevibacter smithii*, within interspecies granules that form in broth culture; the close proximity of the species allows *M. smithii* to benefit from the provision of fermentation products H_2_, formate, and acetate from *B. thetaiotaomicron*, which it uses for methanogenesis.^10^ Meanwhile, *B. thetaiotaomicron's* efficiency of fermentation is inhibited as its products of fermentation accumulate.^10^ Thus, the establishment of a tight syntrophic relationship, where cells of both species are physically situated to allow the transfer of fermentation products from bacterium to archaeon, represents a beneficial relationship that enhances the growth of both taxa.^10^ Notably, this kind of relationship also seems to apply for *M. smithii* with another bacterial species, *Christensenella minuta*, where again a physical association of the two species promotes H_2_ transfer to *M. smithii* from *C. minuta*, while influencing the composition of the fermentation end‐products of *C. minuta* from butyrate to acetate as the majority product.^11^

Examples of production of public commodity substrates are unusual, but known to exist. Perhaps, the best example is of the common gut microbe, *Bacteroides ovatus*, which has been shown to degrade inulin extracellularly, providing the products of this degradation as a commodity for microbes such as *Phocaeicola* (formerly *Bacteroides) vulgatus* while not utilizing inulin or its products as substrates itself. Elegant work has demonstrated that in return, *B. ovatus* receives a fitness advantage from the partner microbe, likely through the provision of a growth‐promoting factor.^12^

Despite the purported long‐term stability of human and animal‐associated gut microbiomes, it is important to point out that these ecosystems can exhibit rapid shifts in composition and function in response to perturbations (e.g., antibiotic introductions or dietary changes).^13^, ^14^ There are also likely daily shifts that are triggered by events such as defecation^15^ and diurnal rhythms.^16^ Thus, ecosystem stability is a function of the frequency at which it is measured, and more work is required to understand and to define ecosystem stability and its relationship to the microbial members that drive it.

### Negative interactions

As alluded to above, ecological theory supports that a stable microbial ecosystem such as the gut microbiome is dominated by negative inter‐microbial relationships, for example, those that involve exploitation and competition, and this is supported by several studies (reviewed in Refs. 5 and 17). Negative interactions in the gut are usually competitive and are categorized as interference or exploitative competition, with the former describing a situation whereby one partner actively damages the other and the latter describing competition between partner species for the same resources.^2^

There are many examples of interference competition between microbes. Certain bacterial secretion systems, most famously the Type VI secretion systems of representatives of the Bacteroidota and Pseudomonadota phyla, employ a retractable spike to inject toxic effector proteins directly from an aggressor cell to the cytoplasm of their victims.^18^ Within the Bacillota phyla, there are analogous examples of this mode of attack, with the use of a Type VII secretion system by some members that exports toxins from the attacking cell to the immediate environment of its victim where they exert bactericidal effects.^19^ Independently of Types VI and VII secretion systems, many bacterial and some archaeal species are known to secrete small, heat‐stable peptides known as bacteriocins, the presence of which is particularly noted in the gut microbiome (reviewed in Ref. 20). Several hundred bacteriocins have been discovered to date, with variable mechanisms of action, including interference with gene expression or protein synthesis, or damage to the bacterial cell wall, and variable spectra of other activities. Prominent examples of bacteriocins secreted by members of the gut microbiome include the colicins produced by Enterobacteriaceae spp. (which also produce neutralizing immunity proteins to protect themselves) and the lantibiotics produced by some Bacillota spp. (e.g., the broad‐spectrum bacteriocin, nisin) that have potent pore formation and cell wall biosynthesis inhibition activities.^20^

Specific bacterial predation is an underappreciated mechanism of interference competition and can be exemplified by certain bacterial species, such as members of the *Bdellovibrio* genus of the Pseudomonadota (Bdellovibrionota) phylum, which have evolved to selectively predate other Gram‐negative bacteria (reviewed in Ref. 21). Most *Bdellovibrio* spp. utilize a highly specialized, obligate mechanism of periplasmic insertion and intracellular development in order to replicate.^22^ Although *Bdellovibrio* spp. are normally associated with the soil environment, a qPCR‐based survey of gut bacterial composition has detected *Bdellovibrio*‐specific genes within human gut samples, interestingly at higher abundance in healthy compared to diseased (inflammatory bowel disease [IBD] and celiac) individuals.^23^ Other predatory activity within the gut environment includes interkingdom interactions, for example, between bacteria and bacteriophages,^24^ and between predatory protozoa and their bacterial prey.^25^ While bacteriophage interactions with their bacterial hosts in the human gut are of enduring interest, the relationship between protozoa and other microbial species of the gut microbiota has, to date, not been deeply explored, perhaps because the presence of protozoa within the gut microbiome is as‐yet underappreciated and seldom measured.

### Exploitation inhibition

Indirect competition for shared resources is usually seen when microbes limit the availability of a given resource to others by consuming it themselves.^2^ This can be done either by employing strategies to more efficiently consume a limiting substrate or by sequestering nutrients. Levy and Borenstein showed, using a metagenomic approach coupled with computational systems biology, that competing species tend to co‐occur within a given niche within the human gut, supporting the process of habitat filtering where coexisting species tend to be more functionally similar than functionally specialized, and thus, exploitation inhibition is likely highly important in shaping the microbiome.^26^ However, there are caveats. For example, Patnode et al. showed in a series of gnotobiotic mouse experiments using tightly controlled dietary substrates that although strains of *B. vulgatus* and *Bacteroides cellulosilyticus* consistently competed for the same niche, *B. ovatus* was able to acclimate to the presence of *B. cellulosilyticus* by being metabolically flexible, highlighting the concessions that some species may make during competitive interactions in order to co‐exist.^27^

Interference and exploitative competition are intrinsically connected within the gut microbiome, and their relative contributions are related to the substrate availability. If resources are plentiful, or competing species density is limited, then exploitation competition dominates; as population density increases, the strategy often shifts toward interference.^28^

To date, most examinations of microbial interactions within the gut microbiome have focused on interactions between individual species. This is through necessity. We first need to understand the range of species–species interactions that might be present in order to then ascertain the relative influence of these interactions as part of a more complex community. There is, however, much more work to be done to measure the complexity and net influences of both positive and negative interactions within a given ecosystem as a whole.

Finally, it is important to remember that the gut microbiome is highly dynamic, and that niches and environmental conditions are constantly shaped according to diet, xenobiotic exposure (not limited to antibiotics), infection, life stage, and lifestyle.^29^, ^30^, ^31^, ^32^ “Bad bugs” are not limited to overt pathogens such as *Salmonella enterica* or *Listeria monocytogenes*, but include pathobionts—members of the gut microbial ecosystem, including *Clostridioides difficile* or *Ruminococcus gnavus*, that are not usually problematic to the host unless there is a shift in the conditions or the population of the ecosystem in their favor.^33^, ^34^ Even classical gut pathogens can be influenced by microbiome members to behave benignly, for example, *S. enterica* and *Vibrio cholerae* have been shown to modulate their virulence traits in the presence of small molecules produced by gut microbial members.^35^, ^36^ Alternatively, the same gut microbiome member may oscillate in its relationship with the host between commensal, mutualistic, and pathogenic states depending on both the host's situation (e.g., the proposed evolution of *Helicobacter pylori* from mutualist to pathogen with host age^37^, ^38^) or its relationship with other microbes (e.g., the interaction between the commensal organism, *Streptococcus cristatus* with the invasive pathogen, *Fusobacterium nucleatum*, that allows the former to hitchhike on the latter and also become invasive to the host).^39^

## MIND YOUR MICROBIOME: MICROBIAL THERAPEUTIC STRATEGIES IN THE CONTEXT OF HUMAN MICROBIAL ECOLOGY

**FIGURE 1:**
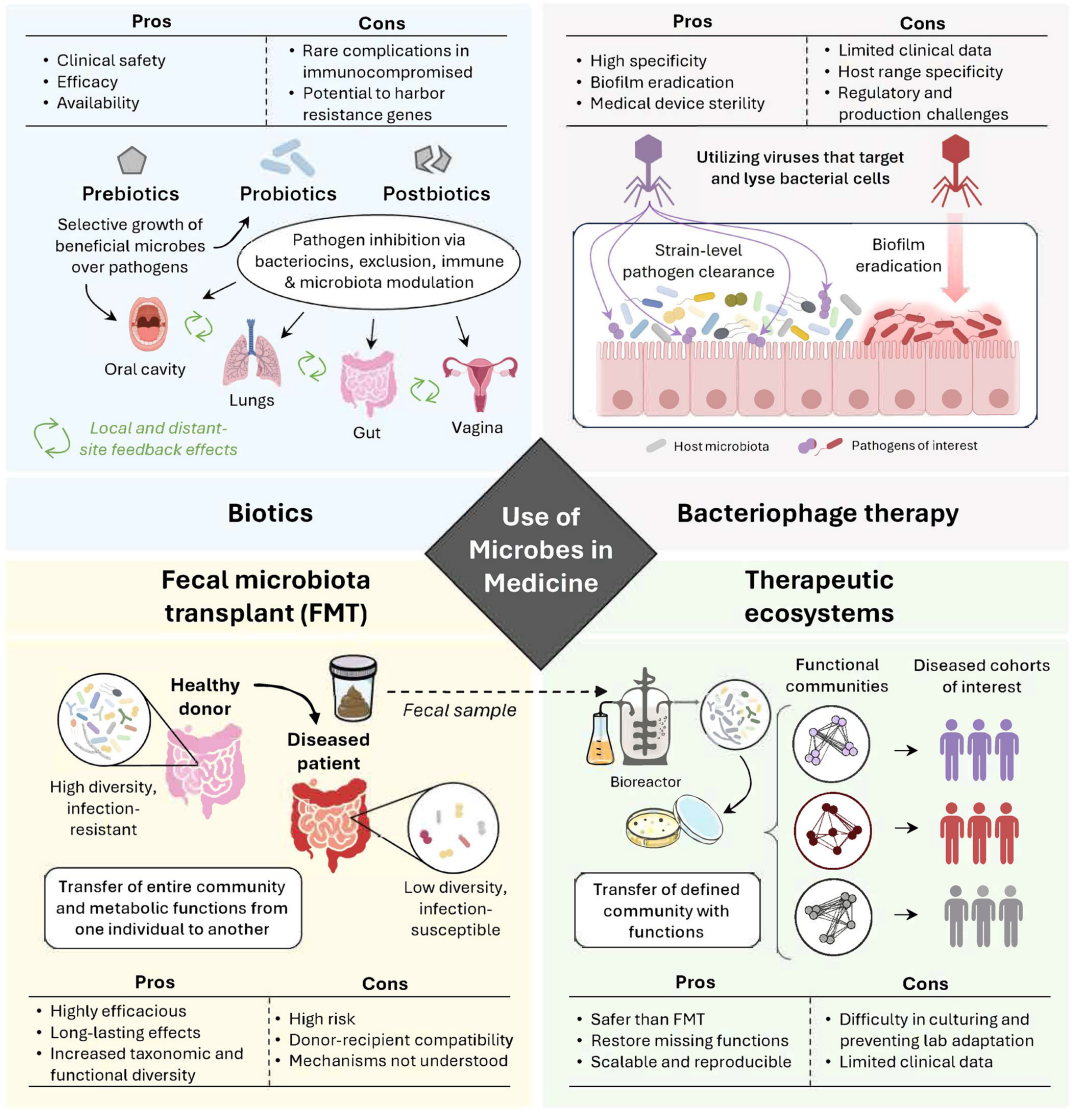
Schematic overview highlighting several types of strategies for the use of microbes as medicine. FMT, fecal microbiota transplant.

In this section, we will describe the current strategies used to attempt to manipulate the gut microbial ecosystem therapeutically, with consideration to the ecological principles outlined in the previous section. An overview schematic is presented in Figure 1.

### Probiotics

Probiotics, defined as “live microorganisms that, when administered in adequate amounts, confer a health benefit on the host,”^40^ boast a historical narrative that extends back to the early 20th century and is grounded in the pioneering explorations of Elie Metchnikoff. A Nobel laureate esteemed as a foundational figure in the field of probiotics, Metchnikoff posited a link between the longevity of Bulgarian peasants and their regular consumption of fermented milk products laden with lactic acid bacteria.^41^ Building on this, contemporary research has elucidated specific mechanisms through which probiotic lactobacilli not only help combat foodborne illnesses^42^, ^43^ but also play a role in the prevention and management of nosocomial infections.^44^, ^45^ These include direct competition for nutrients and binding sites, secretion of antimicrobial substances like bacteriocins, and modulation of the host's immune system to enhance defense mechanisms against pathogens. Nonetheless, a major constraint in the clinical application of probiotics has been the lack of consistent response to therapeutic intervention.^46^

Strain‐specificity is of crucial importance when considering probiotic efficacy from an ecological perspective. For example, human gut‐derived *Lacticaseibacillus rhamnosus* GG is a well‐characterized probiotic strain with strong intestinal adherence properties that are facilitated through its production of three LPxTG‐like surface adhesins. They include MabA,^47^ mucus‐binding MBF,^48^ and SpaCBA pili^49^—the latter of which allows *L. rhamnosus* GG to functionally decolonize opportunistic pathogens (e.g., vancomycin‐resistant *Enterococcus faecium* ^50^) from the intestinal tract by outcompeting them at shared mucosal binding sites.^51^ When compared to dairy‐derived *L. rhamnosus* LC705 lacking a *spaCBA* operon, *L. rhamnosus* GG exhibits over a 40‐fold higher adhesion to human intestinal mucus glycoproteins^52^ and persists significantly longer in the intestinal tract following oral administration.^53^ Consistently, a large‐scale comparative genomics study on 384 *L. rhamnosus* strains revealed a diverse pangenome of at least five distinct phylogenetic clades, with *spaCBA* gene clusters detected in humans—but these were not dairy‐associated strains.^54^ Although colonization is not considered a prerequisite for probiotic efficacy in all cases,^55^ these findings together highlight the importance of considering isolation source and the impact of niche adaptations relevant to strain‐specific interactions with pathogens in a given host.

Mounting evidence also indicates that the efficacy of probiotics is profoundly influenced by the individual's gut microbiota composition, leading to varied responses to identical probiotic strain(s).^32^ Exemplifying this is a large meta‐analysis (*N* = 6261 adults) assessing clinical efficacy of single or mixed culture probiotics against *C. difficile* infection that found a ∼50% response rate across individuals.^56^ Modulation of fecal Clostridiales communities by probiotic intervention with *Lacticaseibacillus paracasei* DG similarly showed mixed responses according to baseline microbiota composition.^57^ In particular, individuals with low initial fecal butyrate levels (<25 mmol/kg of wet feces) experienced over a threefold increase in butyrate concomitant with a 55% reduction in starch‐degrading *Ruminococcus* spp. following *L. paracasei* DG administration, whereas those with high initial fecal butyrate levels (>100 mmol/kg of wet feces) conversely experienced a twofold reduction in butyrate and a concomitant decrease in butyrate‐producing *Faecalibacterium*, *Blautia*, and *Anaerostipes* spp.^57^ Another example is probiotic *Bifidobacterium longum* AH1206, which predictably colonizes ∼30% of individuals based on the presence or absence of endogenous *B. longum* and select carbohydrate utilization genes prior to treatment.^58^ These observations collectively indicate that certain benefits derived from probiotics are intricately linked to the configuration of an individual's microbiome, underscoring both the potential to predict patient response to probiotic interventions, as well as the necessity for future clinical trials to employ statistical methodologies that can account for patient microbiome variations.

Although probiotics offer a promising alternative or complementary approach to traditional antibiotic treatments, it is crucial to acknowledge the potential pitfalls in their use. A key challenge lies in the regulation and quality control of commercial probiotic products. Many probiotic formulations currently marketed as dietary supplements are subject to less stringent regulatory standards compared to pharmaceuticals, leading to concerns about product efficacy, stability, and safety.^59^ Jackson et al. highlighted this issue by showing variability in the quality of probiotic products on the market, including discrepancies between labeled and actual microbial content, which can erode consumer trust and potentially lead to ineffective or unsafe products.^60^

Moreover, recent findings have raised concerns about the consequences of probiotic use, particularly following antibiotic treatment. Suez et al. demonstrated that probiotic administration post‐antibiotics can impair the natural reconstitution of the gut microbiota.^61^ In contrast, autologous fecal microbiota transplantation (FMT) was shown to facilitate faster recovery of the microbiota, suggesting that in some cases, probiotic supplementation may be counterproductive. This study highlights the complexity of microbial interactions in the gut and the need for more personalized approaches when considering probiotic interventions, particularly in the context of gut microbiota recovery post‐antibiotic treatment. Additionally, concerns have been raised regarding the safety of probiotic use in vulnerable populations, such as immunocompromised individuals and premature infants.^62^ Although probiotics are generally considered safe, they have occasionally led to adverse outcomes, including infections caused by the very organisms intended to provide a health benefit.^63^ These risks are compounded by the current regulatory status of probiotics as dietary supplements, which are not subject to the same rigorous safety evaluations as pharmaceuticals.^59^ To mitigate these risks, it is prudent that probiotic preparations, particularly those intended for clinical use, undergo more stringent testing and oversight.

Overall, our growing understanding of probiotics emphasizes the necessity for personalized health strategies, particularly in contrast to traditional antibiotic treatments. Probiotics offer a promising alternative or complementary approach by restoring and maintaining a microbial homeostasis rather than eliminating bacteria indiscriminately as antibiotics do. This nuanced interaction between probiotics and the host's microbiome highlights the potential for reducing antibiotic resistance through targeted microbial interventions. However, the realization of this potential firmly rests on a deeper exploration of the personalized effects of probiotics. Future research is thus imperative, aiming to refine our understanding of when and how probiotics can serve as effective alternatives or adjuncts to antibiotics and tailored to the individual's unique microbiome to enhance therapeutic outcomes and possibly mitigate the global challenge of antibiotic resistance.

### Prebiotics

Prebiotics, defined as “a substrate that is selectively utilized by host microorganisms conferring a health benefit,”^64^ represent another promising therapeutic avenue to either supplement or replace traditional antibiotic strategies. Although many different types of dietary fibers such as pectins, cellulose, and xylans can encourage the growth of a wide range of gut microbes, prebiotics conceptually differ in that their effects are selective. In some cases, prebiotics can antagonize pathogens, as is the case for plantain‐derived nonstarch polysaccharides that have been shown to selectively and dose‐dependently inhibit intestinal adherence of *Salmonella* spp., *Shigella* spp., enterotoxigenic *Escherichia coli*, and *C. difficile*.^65^, ^66^, ^67^ Among prebiotics, inulin, fructo‐oligosaccharides (FOSs), and galacto‐oligosaccharides (GOSs) are the most studied types, with evidence from 30 randomized controlled trials (*N* = 1464 adults) suggesting that an increase in *Bifidobacterium* spp. is the single most common shift observed in bacterial composition.^68^

The bifidogenic effect of prebiotics is particularly intriguing and reflects an eco‐physiological phenomenon. *Bifidobacterium* spp. are dominant in the infant gut microbiota, constituting up to 95% of abundance,^69^ and play a crucial role in the maturation toward a butyrogenic microbiota (phenotypic of healthy adults) via cross‐feeding of substrates to butyrate‐producing *Faecalibacterium*, *Roseburia*, *Anaerostipes*, and *Anaerobutyricum* spp.^70^ Notably, the abundance of *Bifidobacterium* spp. during infancy is attributed to their unique fucosyllactose uptake systems and ability to catalyze human milk oligosaccharides (HMOs), which represent the third most abundant solid component in human milk.^71^ This interaction underscores a co‐evolved mechanism by which maternal milk composition fine‐tunes the neonatal gut microbiota, laying the groundwork for robust immune defenses and disease resistance from early life.^70^

The evolutionary basis of the human diet^72^ may explain why certain health associations with plant‐based foods exist. Dietary polyphenols found in a variety of fruits and vegetables, for instance, have attracted considerable attention due to their antimicrobial, anti‐inflammatory, and anticarcinogenic properties—many of which are now being recognized for their likely microbiota‐mediated effects (reviewed in Ref. 73). From the limited number of clinical trials that have evaluated the prebiotic potential of polyphenols, all suggest that an increase in *Bifidobacterium* spp. is a common response, whereas other taxonomic responses show individual‐ and disease‐specific associations (reviewed in Ref. 74).

Incorporating prebiotics into the diet reflects a profound evolutionary interconnection between humans, their diet, and the microbiome, underscoring a pivotal ecological strategy for enhancing health and bolstering resistance to infectious diseases. This aligns with the hygiene hypothesis, which has been adapted into the biota alteration theory, as described by Villeneuve et al.^75^ This theory suggests that modern environmental changes, including reduced microbial exposure due to sanitized lifestyles, have led to a loss of microbiota diversity, contributing to the rise of chronic diseases. The increasing separation from ancestral microbial exposures has driven interest in strategies to rewild the microbiome.

One such strategy in microbiome rewilding, as proposed by Mills et al., involves reintroducing diverse environmental microbial consortia to restore microbial diversity in human populations.^76^ This approach is particularly relevant in urban settings, where reduced contact with nature and environmental microbes has been implicated in the dysbiosis observed in modern populations. Restoration ecology, through practices like urban habitat restoration, may provide indirect health benefits by reestablishing connections between humans and diverse microbial communities in the environment, potentially enhancing gut and overall health through microbial rewilding. Prebiotics could complement this approach by selectively promoting beneficial microbes that may have diminished in modern lifestyles, thus offering a strategy for both targeted microbial modulation and broader ecological re‐engagement.

An important caveat is that individuals exhibit distinct response patterns to prebiotics, and responder and nonresponder groups are not static due to the acquisition or loss of strain‐level microbiome functions over time.^77^ This suggests that a dynamic approach should be taken, one that focuses on stratifying individuals rather than tailoring prebiotic type to fully leverage as well as delineate the precise role of prebiotics compared to traditional antibiotic therapies. There is also a pressing need for further empirical studies, specifically to directly compare the effects of prebiotics and antibiotics on human health, which would help redefine therapeutic approaches to infectious diseases.

### Synbiotics

Synbiotics are defined as “a mixture comprising live microorganisms and substrate(s) selectively utilized by host microorganisms that confers a health benefit on the host.”^78^ This synergistic approach is a natural extension of the probiotic and prebiotic concepts aiming to introduce beneficial microbes into the gut environment while also providing them with the specific nutrients they need to thrive—ultimately enhancing their ability to colonize and exert positive health effects. A randomized double‐blinded study on formula‐fed infants recently demonstrated that a symbiotic mixture of short‐chain GOSs and long‐chain FOSs (9:1 ratio, respectively) coupled with *Bifidobacterium breve* M‐16 V could effectively develop a gut environment closer to the breastfed reference group.^79^ Another study in adults found that *Bifidobacterium infantis* NCIMB 702205 combined with HMOs led to engraftment in an HMO‐dependent manner, reaching a relative abundance of up to 25% without antibiotic pretreatment or adverse effects.^80^ These studies cumulatively demonstrate the potential of synbiotics to therapeutically establish and reestablish the gut microbiota at different times throughout life.

Current evidence suggests that synbiotics may also help combat antibiotic resistance more effectively than either probiotics or prebiotics alone. For example, *B. breve* 46 and *Bifidobacterium lactis* 8:8 showed strong activity against four strains of *C. difficile* in the presence of GOSs, FOSs, and three other prebiotics.^81^ Similarly, naturally derived prebiotics from garlic and basil significantly enhanced the inhibitory activities of *Pediococcus acidilactici* NCIM 2292.^82^ In terms of clinical findings, a recent meta‐analysis of 35 trials (*N* = 3028 adults) indicated that orally administered synbiotics are associated with a significant reduction in the incidence of surgery‐related complications, including abdominal distention, diarrhea, pneumonia, sepsis, surgery site infection, and urinary tract infection.^83^ These findings should be carefully interpreted though as standard care antibiotics were given in most cases, and there have not yet been any head‐to‐head clinical trials comparing synbiotics to antibiotics for ethical reasons.

At this point, it is also prudent to discuss the importance of considering microbial delivery into the gut. Many pro‐ and synbiotics intended for colonic activity are delivered orally, which creates a technical challenge for manufacturers. Not only do live microbial products need to be protected from the hostile environment of the stomach and proximal small intestine where low pH, digestive enzymes, and high bile salt concentrations limit survival of many microbial taxa,^84^ but the site of microbial release in the gut is also pertinent to its activity. For example, fermentative colonic bacterial species that produce gas as a product of their metabolism may cause discomfort if released prematurely into the small intestine where gas may be trapped.^85^ Prebiotics intended for colonic delivery may result in similar issues if delivered to the small intestine where they may promote blooms of endogenous, gas‐producing microbes normally only present in low abundance. To counteract this, several methods have been developed to encapsulate pro‐, pre‐, and synbiotics. Encapsulation of supplements with pH‐responsive materials such as sodium alginate, or with materials that require degradation by microbes that are found only in the colon, such as chitosan and pectin, is commonly employed by supplement manufacturers. More recently, technologies have extended to micro‐ and nano‐encapsulation using these agents as well as innovative substances such as liposomes and biological membranes (reviewed in Refs. 86 and 87).

Altogether, synbiotics represent a strategic advancement in enhancing gut health and combating antibiotic resistance. This approach has demonstrated significant therapeutic potential, from the replication of the gut environment of breastfed infants to a reduction in postsurgical complications, and highlights its efficacy across various life stages. Evidence of synbiotics’ ability to effectively address pathogen resistance further underscores their value in clinical settings. As research advances, synbiotics are increasingly recognized for their role in personalized medicine, offering a promising avenue for microbiome‐based health interventions and a proactive measure against the global challenge of antibiotic resistance.

### Postbiotics

Postbiotics are defined as a “preparation of inanimate microorganisms and/or their components that confers a health benefit on the host.”^88^ These bioactive compounds, including but not limited to, cell‐free supernatants, peptidoglycans, teichoic acids, and metabolites, have demonstrated significant promise in modulating immune responses, enhancing barrier function, and exhibiting antimicrobial activities (reviewed in Ref. 89). Notably, postbiotics offer distinct advantages over live bacterial preparations such as probiotics or synbiotics.^90^, ^91^, ^92^ For example, postbiotics have potential to be safely utilized in immunocompromised individuals without the risk of infection, combined with antimicrobials without compromising efficacy, and used in regions where stable storage conditions are limited.

Recently, a postbiotic derived from *B. infantis* ATCC 15697 metabolism of breastmilk tryptophan was found to significantly reduce interleukin‐8 response after IL‐1β stimulus in immature enterocytes.^93^ This suggests that a postbiotic approach could be a safe and effective alternative for premature neonates at risk of developing necrotizing enterocolitis.^94^ Several recent reviews have further discussed the potential of postbiotic metabolites such as short‐chain fatty acids that play critical roles in gut health by serving as energy sources for colonocytes, lowering gut pH to inhibit pathogenic bacteria, and modulating inflammatory responses.^95^, ^96^, ^97^

Mechanism studies on postbiotics have been limited to mostly *Lactobacillus* strains, which are a rich source of bacteriocins and other types of metabolites with pathogen‐inhibiting properties.^98^ Focusing on microbiota‐modulating effects, Warda et al. showed that postbiotic Lactobacillus LB—a heat‐treated preparation of biomass and fermentate from *Limosilactobacillus fermentum* CNCM MA65/4E‐1b and *Lactobacillus delbrueckii* CNCM MA65/4E‐2z—could selectively stimulate the growth of *Bifidobacterium* spp. in a fecal fermentation model of the human gut.^99^ This activity was shown to be heat and enzyme stable, not attributed to lactose, and Lactobacillus LB‐like preparations from other commercial probiotics were unable to reproduce bifidogenic effects on the model strain *B. infantis* ATCC 15697.^99^ Dynamic cross‐feeding activities could be one conceivable explanation, although another explanation is that these results serve to highlight the importance of strain‐specificity, reiterating what is known about probiotic mechanisms.

Overall, postbiotics embody a promising frontier in microbiome research and demonstrate the potential to offer targeted, safe, and effective microbial‐based interventions. Their role in health promotion and disease prevention is increasingly recognized, underscoring the need for further research to fully understand their mechanisms of action and optimize their therapeutic applications. As we advance our understanding of the microbiome's complexity, postbiotics may hold the key to unlocking new strategies in managing and preventing a wide array of conditions and complementing the existing microbiome‐modulating approaches of probiotics, prebiotics, and synbiotics.

### Bacteriophage therapy

Bacteriophage therapy, utilizing viruses that specifically target and lyse bacterial cells, offers a highly targeted alternative to conventional antibiotics.^100^ This approach harnesses the natural predatory capabilities of bacteriophages to control pathogenic bacterial populations without affecting beneficial microbiota members.

Two small case studies have so far demonstrated the successful usage of phage therapy in treating multidrug‐resistant *Pseudomonas aeruginosa* in cystic fibrosis patients.^101^, ^102^ Consistently, a larger randomized control trial (*N* = 60) found that custom phage cocktails isolated from various sources (hospital sewage, river Ganga, ponds, and sewers of the Municipal Corporation) were able to sterilize ∼93% of chronic wounds irrespective of age, sex, diabetes status, and infection status by multidrug‐resistant *Staphylococcus*, *Citrobacter, Klebsiella*, and *Proteus* strains.^103^ Research has also explored the application of bacteriophages in biofilm prevention and eradication, which is critical for managing chronic infections and medical device‐associated infections.^104^ Uniquely, phages can lyse bacterial cells within biofilms, having the ability to kill persistent cells and degrade the extracellular matrix via depolymerase activities of virus‐encoded enzymes.^105^ A study on staphylococci biofilms found that the phage lysin LysGH15 not only reduced biofilm mass but also prevented the formation of new biofilms on surfaces, suggesting a therapeutic as well as preventative role.^106^

In terms of treating systemic or intestinal infections with bacteriophages, two recent systematic reviews indicate that there is overall a lack of data to support efficacy.^107^, ^108^ It is thought that phage therapy may be limited within the context of large microbial communities due to rapid selection of phage‐resistant bacteria or immunogenicity as a consequence of prolonged treatment.^109^, ^110^ Nonetheless, preclinical results from mice show promise for orally delivered bacteriophages in curing certain cases of nonalcoholic fatty liver disease (NAFLD) via the targeted decolonization of either high alcohol producing *Klebsiella pneumoniae* ^111^ or cytolysin‐positive *Enterococcus faecalis*.^112^ The exceptional specificity of phages (often targeting only a few strains within a given species unit) is considered a major advantage as it allows elimination of specific pathogens without disturbing other members of the same genus or species.^113^ In cases where this is not favorable though, such as when the host range does not cover all clinically relevant pathogenic strains, tailored engineering of the phage's host recognition domain could represent a solution^114^; multiple clinical trials are underway to evaluate this potential.^115^

Overall, bacteriophage therapy presents a promising and innovative approach to treating bacterial infections that are resistant to traditional antibiotics. With ongoing clinical trials and the development of novel phage‐based technologies, the potential for integrating phage therapy into mainstream medical practice continues to grow, highlighting its role in the future of infectious disease management and microbial ecology. Further research and properly designed clinical studies are essential to address challenges such as phage resistance, regulatory issues, and large‐scale production, ensuring that bacteriophage therapy can be safely and effectively deployed across diverse medical settings.

### Fecal microbiota transplants

FMT, the process of introducing fecal matter from a healthy donor into a diseased patient, is a 1700‐year old medical procedure with roots from 4th century China where it was known as “yellow soup” and used to treat severe diarrhea.^116^ The technique resurfaced in modern medicine in 1958 for the treatment of pseudomembranous colitis,^117^ which ultimately laid the groundwork for FMTs’ later success in treating conditions like *C. difficile* infection.^118^ The utility of FMTs as an alternative to antibiotics is particularly compelling in the context of recurrent *C. difficile* infection (rCDI), for which traditional antibiotic therapies often fail to prevent relapse and can disrupt gut microbiota diversity leading to a vicious cycle of worsening health status. Multiple clinical trials support that FMT can achieve cure rates of ∼80%–100% for rCDI, representing a significant improvement over the ∼20%–30% efficacy observed with vancomycin treatments.^119^ Thus, FMT not only reduces the dependence on antibiotics but also addresses the broader issue of antibiotic resistance by restoring a balanced microbial ecosystem capable of resisting colonization by pathogenic bacteria.

Beyond rCDI, there has been further exploration into FMT's potential for treating a spectrum of other conditions, including IBD,^120^ irritable bowel syndrome,^121^ NAFLD,^122^ neurodegenerative diseases,^123^, ^124^ and several types of chronic metabolic disorders.^125^, ^126^, ^127^, ^128^ Despite its successes, FMT poses a number of risks, including the transmission of infectious agents or harmful genetic material,^129^ and the exact mechanism(s) responsible for beneficial effects largely remain uncharacterized. Moreover, although it is generally accepted that donor selection and sample processing are key determinants of FMT success, one stool does not fit all, and factors affecting recipient engraftment are limited.^130^

One recent study focusing on trans‐kingdom interactions found that the key to successful FMT in rCDI was related to the absence of specific bacteria in donors (*Clostridioides* spp., *Desulfovibrio* spp., *Odoribacter* spp., and *Oscillibacter* spp.) coupled with the absence of fungi (*Yarrowia* spp.) and bacteria (*Wigglesworthia* spp.) in recipients.^131^ However, taxonomic signatures are subject to change depending on disease state. Evaluating the bioenergetic configuration of microbiomes may represent a more generalizable approach, specifically from the perspective of community‐level fitness, cross‐feeding potential, and the degree of redundancy in complementary genetic machinery supporting long‐term stability of microbial networks.^132^ Consistent with this, machine learning on 47,548 genomes from the human gut suggests that implementing an ecology‐based framework is the most effective strategy in understanding strain‐level functions affecting donor strain colonization and recipient strain resilience.^133^

Overall, these findings highlight the transformative potential of FMT in reducing antibiotic reliance and providing alternatives for chronic conditions where traditional drugs fall short. Personalizing FMT protocols to match individual microbiomes could enhance their effectiveness across various diseases. Moreover, integrating detailed microbiome analysis, advanced bioinformatics, and personalized medicine could optimize FMT outcomes, potentially making it a key therapy for a wide range of conditions, including gastrointestinal, systemic, and neurodegenerative disorders. This underscores the need for ongoing research and development to maximize the clinical potential of FMT, ensure safety, and refine treatments for broader use in modern medicine.

### Therapeutic microbial ecosystems

Therapeutic ecosystems represent an amalgamation of probiotics and FMT, combining the enhanced safety and reproducibility of utilizing pure, well‐curated strains with augmented microbial diversity. The earliest mainstream medical use of therapeutic ecosystems was by Tvede and Rask‐Madsen,^134^ who were the first to successfully use a purified combination of 10 stool‐derived bacterial strains to treat 6 patients with pseudomembranous colitis caused by *C. difficile* infection. At the time, the approach did not take off, likely because of the difficulties of culturing fastidious anaerobes, combined with the relatively easy access to effective antibiotics for treating this infection. However, decades later, increasing reports of rCDI that could not be effectively managed by antibiotic treatment alone drove a resurgence of interest in FMT, as mentioned above, and by extension, an interest in developing safer microbial cocktails that would be more readily available than stool.^135^ Several examples of microbial ecosystems created for clinical use, designated as live biotherapeutic products (LBPs), have been reported in the recent literature and are briefly discussed below.

A mixture of 33 purified bacterial strains isolated from a single healthy donor was successfully used to treat two patients with rCDI through administration via colonoscope,^136^ with the approach then further developed through pharmaceutical company partnership (NuBiyota with Takeda Pharmaceuticals) into a series of ecosystems, each containing ∼30 bacterial species designed for oral delivery and for several indications, including rCDI,^137^ depression and anxiety,^138^ and adjunct immunotherapy in oncology.^139^ Efficacy in rCDI treatment was demonstrated, whereas to date, only dose‐ranging/tolerability trials have been completed for this product for other indications.

Vedanta Biosciences has reported on the development and clinical use of an orally delivered, eight‐species mixture of *Clostridium* spp. representative of three separate clades (VE303). This group first showed that this product could colonize healthy volunteers following pre‐treatment with vancomycin^140^ and then went on to demonstrate clinical efficacy for the treatment of rCDI in a small, dose‐ranging study.^141^

SER‐109 is an example of a purified product from donor stool which, although it is not representative of axenically cultured strains, still falls into the LBP category. SER‐109 (since brought to market as VOWST) features a purified preparation of bacterial endospores from donor stool.^142^ Although only a relatively small fraction of the total human gut microbiome diversity is representative of species that can form endospores^143^ (thus limiting the overall species diversity of the product), VOWST has been shown to be safe and moderately effective for the treatment of rCDI.^144^ Interestingly, both VOWST and MET‐2 (a NuBiyota ecosystem used to treat rCDI) have shown, through post hoc analyses of clinical trial data, added benefits of reducing both the abundance of Pseudomonadota spp. (a general marker of ecosystem dysfunction^145^) and antimicrobial‐resistant organisms in patient stool samples collected post therapy.^146^, ^147^ These changes suggest the displacement of antimicrobial‐resistant microbes through competition with strains of greater fitness, the refilling of vacant niches affected by therapy, or both.

The efficacy of these LBPs was measured by the developers in terms of engraftment, where taxonomic sequence analysis was used to measure the number of species present before and after treatment and compare this to the sequences of the therapeutically introduced strains.^137^, ^144^ Although it seems likely that this measurement would directly assess the ability of the delivered microbes to colonize, the presence of the same or similar endogenous species in a given patient with a rebound in their numbers following treatment cannot be excluded. Nevertheless, it seems clear that treatment of patients with rCDI with therapeutic ecosystems effects at least a transient, significant change in ecosystem composition and function that correlates with patient outcomes.^137^, ^144^, ^148^

The use of pure bacterial preparations as LBPs, while steeped in clinical potential, may also present an ecological challenge. For example, Wilde et al. recently showed that bacterial isolation from stool samples followed by purification and subsequent coculture as a defined community specifically selected against virulent bacteriophages.^149^ Drawing on the discussion at the start of this review with regard to the factors that shape gut microbial ecosystems, it is likely that such virulent bacteriophages influence microbial ecology in the niches of the gut in ways that are as‐yet underappreciated, suggesting that future LBPs might benefit from the deliberate addition of predatory elements such as virulent bacteriophages (or *Bdellovibrio* spp., mentioned above).

In general, the development of pure LBPs from gut‐derived microbes has suffered from a lack of investment coupled with stringent regulatory hurdles; for example, MET‐2 is manufactured by separately culturing pure strains of component bacteria with each component considered as a distinct LBP for quality assurance purposes. This manufacturing process greatly adds to the cost of production. The result is that, to date, only two LBPs have reached the market—VOWST and an FMT product marketed as REBYOTA.^150^ Although both these products have shown efficacy in the treatment of rCDI, they are derived from stool and thus there will always be some ambiguity about the microbial strains they contain, potentially compromising their effectiveness.

## WHERE IS THE FIELD GOING?

### Next‐generation probiotics (NGPs): engineered and nonconventional probiotics

Engineered probiotics refer to probiotic strains that have been genetically modified in order to enhance their health‐promoting attributes (reviewed in Refs. 151 and 152). In reductive cases, engineered probiotics may be strains that have beneficial potential, but which carry undesirable attributes such as carriage of genes for antimicrobial resistance or broad‐acting bacteriocins; such strains may be genetically engineered to remove these troublesome features to improve strain safety profiles.^153^ In additive cases, engineered probiotics may represent strains which have been modified to provide them with an additional, beneficial, or useful characteristic to target a select situation. For example, a *Lacticaseibacillus casei* strain was bioengineered to express an adhesin from a nonpathogenic *Listeria* strain that enabled the modified organism to outcompete the colonization of pathogenic *L. monocytogenes* in a mouse model of infection.^154^ The advent of technologies for precise gene editing, such as CRISPR‐Cas9, has fueled interest in bioengineering approaches to improve probiotic strains and to create novel strategies to diagnose and treat disease.^153^ As an example, the probiotic *E. coli* strain, Nissle 1917, is easily transformable using a wide array of genetic tools and has acted as a chassis for several innovative approaches for treatment of specific metabolic diseases such as phenylketonuria (through the addition of a phenylalanine metabolizing enzyme),^155^ enteric hyperoxaluria (through the addition of combined oxalate metabolizing genes from *Oxalobacter formigenes* and *Saccharomyces cerevisiae*),^156^ and hyperammonemia^157^ (through overproduction of arginine to sequester excess ammonia). These engineered probiotics have had mixed success in human clinical trials.^155^, ^156^, ^157^ In in vitro and in vivo (mouse) models, *E. coli* Nissle 1917 has also been used to bioengineer strains which can: (a) sense tetrathionate and produce an antimicrobial to inhibit salmonella infection,^158^ (b) express genes encoding gamma‐aminobutyric acid to ameliorate neurotransmitter deficiency,^159^ and (c) produce high levels of l‐arginine to activate an effective antitumor response by T cells.^160^

One of the major concerns about the use of genetically modified organisms such as NGPs in medicine is the potential for strains to escape and contaminate the environment. Concerns relating to this issue can be mitigated through the incorporation of biocontainment strategies. For example, NGPs can be engineered to repress an essential gene on entering a particular set of environmental conditions (such as those found in the gut), or NGP strains can be engineered to be auxotrophic for particular essential nutrients that are not available in the target system, such that they can only be cultured when this auxotrophy is relieved in a laboratory setting (reviewed in Ref. 161). In reality, redundancies should be built into these kinds of biocontainment strategies to prevent escape due to random mutations. Despite the promise of precise engineering approaches to create new medicinal probiotics, very little work has been done to assess the effects of such strategies on the ecology of the microbiome, although it would seem that this is an essential part of any safety assessment. It is telling that although the risks and benefits of probiotic use in different populations have been clearly outlined,^162^ global regulatory agencies seem to have no unified approach to the oversight of genetically modified NGPs.

In the wake of the difficulties in the commercialization of engineered NGPs, nonconventional probiotics are gaining momentum as naturally occurring alternatives. Generally speaking, most currently available, clinically validated probiotic strains currently on the market are representatives of a very small number of microbial taxa compared to the diversity seen in the human microbiome. Recognizing this, and also that several bacterial taxa from the human microbiome (and particularly the gut) have recently been appreciated for widely beneficial attributes, attention is now turning toward novel strains from such taxa. Examples include *Akkermansia muciniphila* that has been shown to increase glucagon‐like peptide‐1^163^ and to regulate lipid homeostasis,^164^ several butyrate‐producing species such as *Faecalibacterium prausnitzii* whose butyrate production can ameliorate inflammation through the inhibition of the NF‐kappaB pathway,^165^ and *Anaerobutyricum* (formerly *Eubacterium) hallii*, which can transform and thus detoxify a prevalent dietary carcinogen (heterocyclic amine 2‐amino‐1‐methyl‐6‐phenylimidazo[4,5‐*b*]pyridine).^166^ The safety profile of many such beneficial microbes from the human gut has been boosted by the use of both FMT and therapeutic microbial ecosystem strategies to treat disease (discussed above), which have generally demonstrated excellent safety and tolerability.^137^, ^138^, ^139^, ^140^, ^141^, ^142^

Despite the difficulties encountered in transforming members of the gut microbiome (e.g., many of these species are nutritionally fastidious and strictly anaerobic, thus difficult to culture), there has been a recent, concerted effort by researchers to develop new molecular tools to edit and manipulate gut microbial strains through gene‐deletion and gene‐swapping strategies in order to understand their ecological roles. For example, the genes for the complete production pathways for deoxycholic and lithocholic acids (DCA and LCA, respectively) from *Clostridium scindens* were cloned into a strain of the non‐DCA and LCA‐producer, *Clostridium sporogenes* to demonstrate an approach to control the bile acid pool within the microbiome.^167^ The success of this effort (recently reviewed in Ref. 168) will undoubtedly fuel the future of the field. The use of therapeutic, genetically modified strains that originated in the gut niche, for example, holds a key ecological advantage over traditional probiotic strains in terms of their abilities to survive, adapt, and interact with other microbes within the environment in which they first evolved.

### “Missing microbes” and their potential

Over the last decade, several metagenomic studies on the gut microbiomes of people living in different regions of the world have revealed striking patterns of diversity.^169^ Specifically, individuals living within societies that practice traditional hunter‐gatherer lifestyles reminiscent of those thought to be widespread before the advent of the Industrial Revolution were shown to have far higher microbial diversity than those living in an urbanized environment.^170^, ^171^, ^172^ In 2009, Blaser and Falkow put forward their “missing microbiome” hypothesis that posited that microbial ecosystem diversity loss in the human microbiome was a result of extinction events over generations through modern lifestyle practices such as antibiotic use and changes in dietary intake.^173^ This theory was bolstered by the work of Wibowo et al., who reconstructed metagenome‐associated genomes from DNA recovered from human paleofeces samples between 1000 and 2000 years old, demonstrating that these microbial populations were more similar to those of modern nonindustrial populations than modern industrial populations.^174^ Fragiadakis et al.^175^ refer to taxa that seem to have been lost or rarified within the microbiomes of individuals living in urbanized environments as VANISH taxa (volatile and/or associated negatively with industrialized societies of humans), and these taxa have been found within several geographically distant hunter‐gatherer communities, as well as paleofeces, suggesting that urbanization may indeed be the culprit behind this loss in diversity.

Based on these findings, as well as studies in laboratory mice that suggest that rewilding the animals’ microbiomes with microbes derived from wild‐caught mice or obtained naturally through the environment might induce increased (and overall beneficial) immune activation in these animals,^176^ attention in human medicine has been given to the concept of rewilding the human microbiome. Blaser describes the concept of rewilding the microbiome as a process involving first the removal of opportunistic species that currently inhabit vacated niches and then replacing these with the VANISH taxa that presumably first occupied them.^177^

VANISH taxa include species belonging to the bacterial families Prevotellaceae, Succinivibrionaceae, Paraprevotellaceae, and Spirochaetaceae^175^ that are likely to be particularly specialized to elements of the hunter‐gatherer diet, such as a very high intake of dietary fiber. Recently, for example, *Treponema peruense* was described as a novel taxon derived from stool samples taken from members of a remote community in the Peruvian Amazon; the genome of this strain of species was found to be rich in genes predicted to be used in carbohydrate and amino acid transport and metabolism, although metabolites beyond the major fermentation end products were not explored.^178^ *Prevotella copri* strain genomes are also rich in polysaccharide utilization loci and the presence of this species has been associated with improvements in weight gain in malnutrition^179^ and glucose metabolism in healthy individuals,^180^ but also an increased incidence of obesity in otherwise healthy children.^181^ The presence of *P. copri* is also associated with inflammatory conditions such as ankylosing spondylitis and rheumatoid arthritis.^182^, ^183^ In general, VANISH taxa include species that are nutritionally fastidious and difficult to culture, and combined with the bioethical considerations that accompany research with indigenous communities, little has been done to date to establish culture collections from hunter gatherers.

There is debate in the literature as to whether rewilding the human microbiome is likely to be of benefit anyway, considering that maintenance of many of these microbes, if they could be developed into therapeutics, would require a considerable dietary change.^184^ Instead, rejuvenation of the microbiome, for example, using autologous, stored feces banked during healthful youth to restore one's own microbiome later in life has been proposed as an alternative and likely is a more useful strategy.^184^ However, exploration of VANISH taxa as potential novel nonconventional probiotics, either as individual strains or as microbial consortia, still holds a great deal of potential for the future of the field.

### Personalized microbiome modulation

It has long been established that, despite some level of functional redundancy between individuals, the microbiome composition is unique to its host, and relatively homeostatic. Elegant work by Chen et al.^185^ who used a metagenomic approach to determine how the gut microbiome changed over time (up to 4 years) demonstrated a level of stability in microbial composition that was robust enough to allow molecular fingerprinting to differentiate and discern hosts, even though member species tended to show variations in temporal genetic stability. Because of this, there has been recent interest in the concept of personalized medicine as it relates to the microbiome. Microbiome‐derived data have been shown to correlate with disease states, providing opportunities to develop novel screening methods for conditions such as Type 2 diabetes and Alzheimer's disease.^186^, ^187^ But beyond this, as new knowledge of microbiome function is gained, this opens up an exciting new avenue for control and/or prevention of disease through individualized modulation of the microbiota.

It has been clearly shown that different individuals respond physiologically to the same food substrates in diverse ways; for example, the glycemic index of foods varies across individuals and is associated with gut microbiome composition.^188^, ^189^, ^190^ This, in turn, demonstrates the feasibility of personalized nutrition as a strategy to work with a patient's microbiome to improve health outcomes. Indeed, a machine‐learning algorithm that utilized microbiome features as a component of the input data has been used in a clinical trial to assess the effect of personalized nutrition on postprandial glycemic response, with the results ultimately challenging the utility of measuring the carbohydrate content of a meal to predict this response.^189^ In addition, microbiome profiles may help to predict individual responses to xenobiotics such as food additives,^191^ antibiotics, or other drugs^192^, ^193^—and even vaccines.^194^

Despite the promise of personalized microbiome modulation, the field is currently in its infancy, with the major barriers to its development being the cost and difficulty of deep individual microbiome assessment at a population scale, coupled with the challenges of deciphering cryptic microbial genomes and their functions, and determining causality over correlation of microbiome elements in host health (reviewed in Ref. 195). However, multi‐omics integration approaches to understanding the microbiome continue to evolve at a rapid pace, such that it may well soon be commonplace for medical professionals to prescribe drugs that consider patient microbiome health and composition, and for the general public to shop for groceries labeled to indicate specific benefits toward particular microbial profiles.

## CONCLUSIONS AND OPEN QUESTIONS

The field of human microbiome science is evolving rapidly and intertwining increasingly with medical research. Traditional probiotic strains, while valuable for augmenting or replacing antibiotics in some instances, are not one‐size‐fits‐all solutions and are being supplanted by more strategic approaches to modulating gut health: from using novel strains with specific beneficial properties as next‐generation probiotics, to incorporating strategies to specifically remove problematic microbes or enhance the beneficial effects of endogenous species, to replacing or augmenting microbiomes with entire ecosystems. Beyond the global issue of antibiotic resistance, there is a growing understanding of the detrimental impacts of antibiotics on our microbiomes, providing the impetus to discover novel strategies to treat disease. Nevertheless, significant challenges remain such as the extensive interindividual variability in microbiome composition and our limited understanding of the majority of microbes that constitute the human microbiome. Fortunately, advancements in omics technologies and bioinformatics are starting to yield fruit, making microbiome research one of the fastest developing areas in the biological sciences today. Below, we highlight several key unresolved questions in the field:

- What is the level of bacterial and archaeal interspecies strain diversity amongst individual microbiomes in both healthy and diseased persons? Are accessory genomes among strains within a given species infinite or limited, and what are the drivers and constraints? How does strain diversity develop over a person's lifetime and what are the consequences?
- How do interactions between single microbial species within an ecosystem influence the overall ecosystem dynamics? What interactions are most important, and which would have the most influence as targets for intervention or modulation?
- How can we best tackle the problem of understanding microbiome *dark matter* (i.e., genetic material for which we currently have no frame of reference for identification and characterization)? How can we do this at a population scale while considering trans‐kingdom interactions of novel bacteria, archaea, fungi, and protozoa?
- How can we develop personalized microbiome therapeutics on a large scale at an affordable cost? Can people be divided into groups based on shared microbiome properties that could be targeted by modulation therapies? How can we analyze clinical data in a statistically robust way that effectively considers microbiome property differences between responders and nonresponders?
- What are the long‐term effects of microbiome modulation? With enough application, can microbiomes be modulated permanently? How does supplementation with microbiome‐based therapeutics affect the immune system? Are there unforeseen risks of microbiome‐directed therapy?
- Can an insufficiency in the microbiome be rectified through dietary manipulation alone? How much does horizontal acquisition of microbes contribute to changes in the microbiome during different life stages?
- What clinical situations can microbial therapeutics be most successfully applied to? While treatment of *C. difficile* infection using microbial therapeutics has been largely effective, what other diseases are likely to see benefits through this approach? How can successes seen in the research laboratory be most effectively transferred to the clinic?
- How can we develop a unified regulatory system for the development of novel microbiome therapeutics that both ensure safety and efficacy and promote innovation? How can microbiome modulation be accurately assessed? Can genetically modified microbes be safely incorporated into novel therapeutics, and what are the risks and consequences of environmental escape?
- Can we define a healthy microbiome? To what degree are hunter‐gatherer microbiomes considered healthy? And which components, and functions, are missing in urbanized societies? How can we ethically source missing microbes?
- What technological developments are required to better equip microbiome researchers to understand host–microbiome interactions? Can advances in artificial intelligence (e.g., natural language processing and deep generative learning models) be exploited to further our understanding of microbiome dynamics and host–microbiome interactions?

## AUTHOR CONTRIBUTIONS

**Brendan A. Daisley**: Writing—original draft; writing—review and editing. **Emma Allen‐Vercoe**: Conceptualization; writing—original draft; writing—review and editing; supervision; and funding acquisition.

## CONFLICT OF INTEREST STATEMENT

E.A‐V. was co‐founder of NuBiyota, a company developing therapeutic microbial ecosystems for treatment of human diseases.

### PEER REVIEW

The peer review history for this article is available at: https://publons.com/publon/10.1111/nyas.15237
